# Time trends in the incidence of clinically diagnosed type 2 diabetes and pre-diabetes in the UK 2009–2018: a retrospective cohort study

**DOI:** 10.1136/bmjdrc-2020-001989

**Published:** 2021-03-19

**Authors:** Kingshuk Pal, Laura Horsfall, Manuj Sharma, Irwin Nazareth, Irene Petersen

**Affiliations:** Research Department of Primary Care and Population Health, University College London, London, UK

**Keywords:** epidemiology, diabetes mellitus, Type 2, prediabetic state

## Abstract

**Introduction:**

To describe recent trends in the incidence of clinically diagnosed type 2 diabetes and pre-diabetes in people seen in UK general practice.

**Research design and methods:**

A retrospective cohort study using IQVIA Medical Research Data looking at people newly diagnosed with type 2 diabetes and pre-diabetes through primary care registers in the UK between 1 January 2009 and 31 December 2018.

**Results:**

A cohort of 426 717 people were clinically diagnosed with type 2 diabetes and 418 656 people met the criteria for a diagnosis of pre-diabetes in that time period. The incidence of clinically diagnosed type 2 diabetes per 1000 person years at risk (PYAR) in men decreased from a peak of 5.06 per 1000 PYAR (95% CI 4.97 to 5.15) in 2013 to 3.56 per 1000 PYAR (95% CI 3.46 to 3.66) by 2018. For women, the incidence of clinically diagnosed type 2 diabetes per 1000 PYAR decreased from 4.45 (95% CI 4.37 to 4.54) in 2013 to 2.85 (2.76 to 2.93) in 2018. The incidence rate of pre-diabetes tripled by the end of the same study period in men and women.

**Conclusions:**

Between 2009 and 2018, the incidence rate of new clinical diagnoses of type 2 diabetes recorded in a UK primary care database decreased by a third from its peak in 2013–2014, while the incidence of pre-diabetes has tripled. The implications of this on timely treatment, complication rates and mortality need further longer term exploration.

**Significance of this study**

**What is already known about this subject?**

- Previous studies have shown various trends in different countries suggesting that the incidence of type 2 diabetes might be stabilizing or falling.
- There are little data about trends in clinical diagnoses of type 2 diabetes since the diagnostic criteria changed in 2011 to allow diagnosis based on HbA1c levels.

**What are the new findings?**

- The incidence rate of new diagnoses of type 2 diabetes recorded in primary care records in the UK has dropped by a third since 2013, while the incidence rate of pre-diabetes has tripled.
- More people in the UK are now being diagnosed with pre-diabetes than type 2 diabetes.
- Rates of diagnosis of type 2 diabetes appear to have fallen more in older age bands compared with people aged 40–49.

**How might these results change the focus of research or clinical practice?**

- Further research is needed to understand if the current single threshold for HbA1c for diagnosing type 2 diabetes is appropriate and to understand the implications for the risks in those increasingly being diagnosed with pre-diabetes.

## Introduction

Type 2 diabetes is a growing health problem across the world, affecting over 400 million people and with estimates that it could affect nearly 700 million people by 2045.^1^ In the USA, the prevalence of diabetes is estimated to be between 12% and 14% with a further 38% of the population at high risk of developing diabetes.^2^ In the UK, the prevalence of type 2 diabetes doubled between 2000 and 2010 to 5%.^3^

Diabetes is associated with renal failure, blindness and peripheral vascular disease and the higher risks of myocardial infarction, strokes and other fatal complications can shorten life expectancy by 8–10 years if diabetes is poorly controlled.^4^ Worldwide, over 500 billion dollars is spent on treating diabetes and most is spent on treating diabetes related complications.^1 5^

In the UK, spending on diabetes and related complications accounts for nearly 10% of the total National Health Services (NHS) budget.^5 6^ Changes in the incidence and prevalence of type 2 diabetes will have significant implications for healthcare services like the NHS. A recent systematic review found evidence of different trends in incidence across the world but described a stable or decreasing incidence in a most studies.^7^ In the UK, increasing incidence has been observed until 2010 but there are little data on trends over the last decade.^3 8^

Closely linked to type 2 diabetes is a metabolic state that lies between normal glucose homeostasis and type 2 diabetes, which has been defined as pre-diabetes.^9^ People with pre-diabetes are at high risk of developing type 2 diabetes, with 5%–10% of people progressing to diabetes per year and evidence of early diabetes related complications.^10–13^ Definitions of pre-diabetes include people with impaired fasting glycemia, impaired glucose tolerance and HbA1c levels below the threshold for diagnosing type 2 diabetes.^14–16^ The prevalence of pre-diabetes in adult populations is on the rise and estimated at 35% in the UK and USA and as high as 50% in China.^17^ Diabetes and pre-diabetes are part of a spectrum of metabolic disorders that overlap significantly. The main purpose of this study was to examine the trends in incidence of type 2 diabetes and pre-diabetes as recorded by the family physician (general practitioners (GPs)) in electronic health records for people seen in UK general practice over 10 years from 2009 to 2018.

## Methods

### Data source

This was a retrospective cohort study using data from the IQVIA Medical Research Data (IMRD)-UK data. This contains electronic primary care health records for approximately 12 million individuals in the UK from more than 700 general practices. Multiple validation studies have shown IMRD data to be broadly generalizable to the wider UK population.^18–20^ IMRD contains records from routine consultations in primary care with details of medical conditions, symptoms, diagnoses and prescriptions issued by GPs. A hierarchical recording system of Read codes has been used to classify symptoms and diagnoses.^21^ In addition, the database includes Townsend scores as a measure of social deprivation.^22^ Social deprivation is assigned quintiles with 1 being the least deprived and 5 being the most. The majority of diabetes care in the UK is provided through primary care and GPs are incentivized to maintain registers of people with diabetes, which encourages coding of clinical data. IMRD data are therefore likely to represent a comprehensive record of routine diabetes care in the UK. Data have been reported in line with STROBE guidance for describing cohort studies.^23 24^

### Definitions

People living with type 2 diabetes were identified using a previously published algorithm.^3^ Individuals were diagnosed with diabetes if they had at least two of the following records: (1) a diagnostic code for diabetes, (2) supporting evidence of diabetes, for example, two raised HbA1c levels above 7.5% (48 mmol/mol) or screening for diabetic retinopathy or (3) treatment for diabetes. The Read codes used can be found in Appendix 1 (online supplemental file). The first record of any of these three was considered as the date of diagnosis. Records with Read codes for maturity onset diabetes of the young, latent autoimmune diabetes of adulthood, polycystic ovarian syndrome or just gestational diabetes were not included in the cohort for type 2 diabetes. People with Read codes for type 1 diabetes and those under 35 who had only ever been prescribed insulin were not included in the cohort of people with type 2 diabetes as they were likely to have type 1 diabetes.

10.1136/bmjdrc-2020-001989.supp1 Supplementary data

People with pre-diabetes were identified using either the Read codes for impaired fasting glycemia, impaired glucose tolerance and pre-diabetes listed in Appendix 1 (online supplemental file) or an HbA1c level of 6.0%–6.4% (42–47 mmol/mol). Records with Read codes for maturity onset diabetes of the young, latent autoimmune diabetes of adulthood or polycystic ovarian syndrome were not included in the pre-diabetes cohort. Patients who subsequently met the diagnostic criteria for type 2 diabetes were included in the cohort up to the point of a clinical diagnosis of type 2 diabetes.

### Study population and period

Data from general practices contributing data to IMRD between 1 January 2009 and 31 December 2018 were used for this study. Data quality was improved by using practices which had reached the standard for acceptable computer usage and mortality reporting.^25 26^ For inclusion in the cohort for incidence, we included individuals who had at least 9 months of data available. Individuals were followed up from the latest of 9 months after they registered with the GP practice or the date when the practice provided data that met the quality criteria set out above. People who had been registered for less than 9 months at the practice prior to diagnosis were excluded from the incident cohort as they were more likely to represent prevalent cases.^3^ Follow-up time continued until the earliest of: death, date of leaving the practice, the practice stopped contributing data or date of diagnosis with type 2 diabetes.

### Analyses

The incidence of type 2 diabetes was estimated per 1000 person years at risk (PYAR). This was calculated by dividing the number of new cases diagnosed over the study period by the total follow-up time for people at risk of developing type 2 diabetes in that period, multiplied by 1000. We determined incidence rates by age, gender, social deprivation (Townsend Score) and calendar year. In considering the follow-up time for our denominator, we censored follow-up when patients died or left the practices. Likewise, we calculated incidence rates for pre-diabetes but excluded those with a clinical diagnosis of type 2 diabetes from the date of their diagnosis of diabetes. A negative binomial regression model was used to estimate changes in incidence by age, gender, social deprivation and calendar year while adjusting for the other respective variables.

Analyses were conducted with Stata software V.16.0 (Stata, USA).

## Results

**Figure 1:**
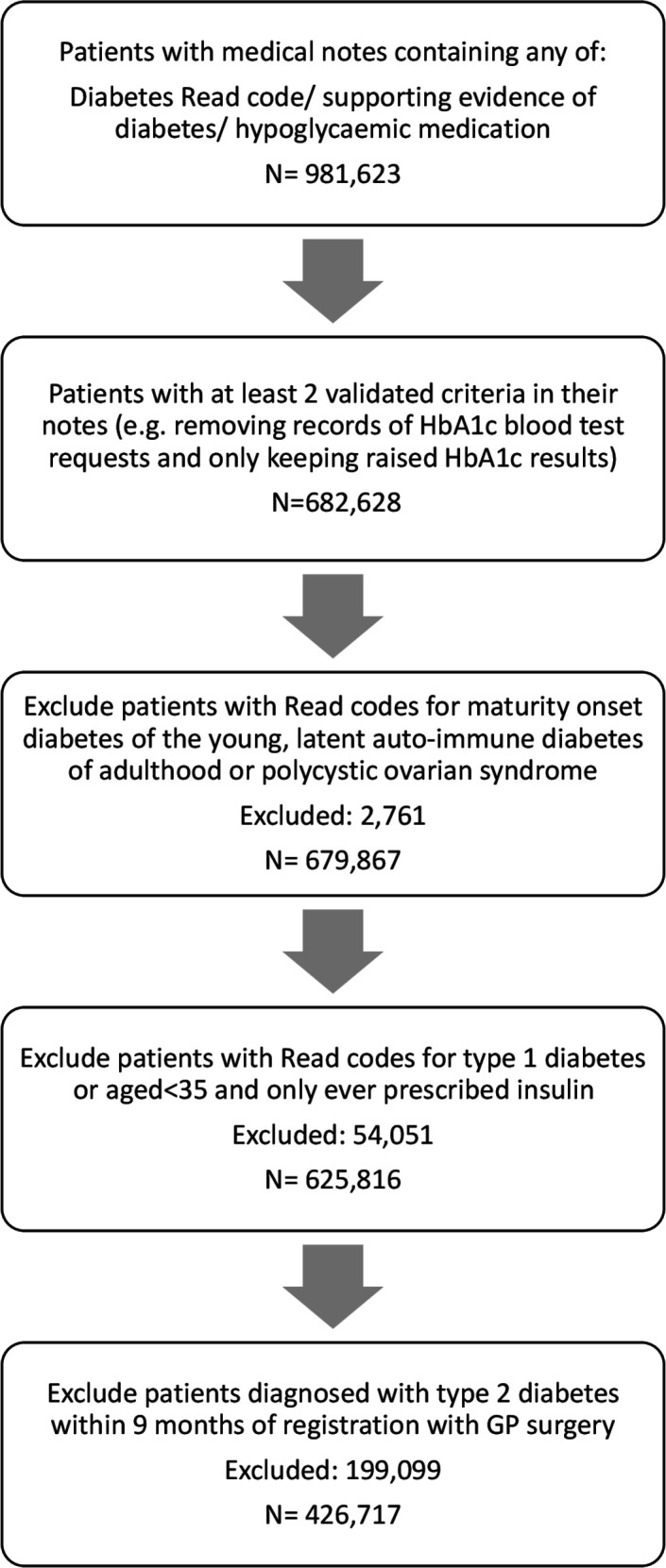
Flowchart for patients included in type 2 diabetes cohort.

In total, 625 816 individuals with type 2 diabetes were identified in the study, of whom 426 717 (70%) were newly diagnosed between 1 January 2009 and December 2018 (figure 1). The baseline characteristics of the cohort can be found in table 1. Just over half (53%) of the cohort were men. The mean age of diagnosis was 60.4 in men and 61.7 in women. In addition, 418 656 people met the criteria for a diagnosis of pre-diabetes during this period.

**Table 1:** Baseline characteristics of included patients

Characteristic	Type 2 diabetes	Pre-diabetes
N	426 717	418 656
Women	198 683 (47%)	212 649 (51%)
Mean age at diagnosis in years (SD)	Men: 60.4 (13.2) Women: 61.7 (15.3)	Men: 62.8 (13.7) Women: 64.4 (15.4)
Mean BMI within 2 years of diagnosis (SD)	Men: 30.9 (5.8) *Missing: 19 725* (*9%*) Women: 32.0 (7.4) *Missing: 18 633* (*9%*)	Men 29.1 (5.5) *Missing 64 709* (*31%*) Women 29.5 (6.9) *Missing 55 987* (*26%*)

BMI, body mass index.

### Incidence of type 2 diabetes

The overall incidence of recorded type 2 diabetes in men was 4.51 (95% CI 4.49 to 4.53) per 1000 PYAR while in women, it was 3.88 (95% CI 3.86 to 3.90) per 1000 PYAR (table 2). The adjusted incidence risk ratio (IRR) for women, compared with men, was 0.86 (95% CI 0.85 to 0.87).

**Table 2:** Incidence of type 2 diabetes by age and deprivation

	Rate per 1000 PYAR (95% CI)		Adjusted IRR (95% CI)*	
	Men	Women	Men	Women
Overall	4.51 (4.49 to 4.53)	3.88 (3.86 to 3.90)	1	0.86 (0.85 to 0.87)
Age, years				
0–19	0.09 (0.08 to 0.09)	0.15 (0.14 to 0.16)	0.02 (0.02 to 0.02)	0.05 (0.04 to 0.05)
20–29	0.40 (0.38 to 0.41)	0.92 (0.89 to 0.95)	0.09 (0.08 to 0.09)	0.27 (0.26 to 0.28)
30–39	1.47 (1.44 to 1.51)	1.69 (1.66 to 1.72)	0.34 (0.33 to 0.35)	0.52 (0.51 to 0.54)
40–49	4.28 (4.23 to 4.33)	3.16 (3.12 to 3.21)	1	1
50–59	8.31 (8.23 to 8.39)	5.82 (5.75 to 5.88)	1.98 (1.95 to 2.01)	1.89 (1.85 to 1.92)
60–69	12.49 (12.38 to 12.60)	8.85 (8.76 to 8.94)	2.98 (2.94 to.3.03)	2.87 (2.82 to 2.92)
70–79	13.69 (13.54 to 13.84)	11.01 (10.89 to 11.13)	3.28 (3.23 to 3.34)	3.55 (3.50 to 3.63)
80–89	10.55 (10.35 to 10.76)	8.86 (8.72 to 9.00)	2.52 (2.47 to 2.58)	2.82 (2.76 to 2.89)
90–99	7.02 (6.58 to 7.48)	5.39 (5.16 to 5.63)	1.69 (1.58 to 1.80)	1.74 (1.67 to 1.83)
Townsend quintile				
1	4.18 (4.13 to 4.22)	3.20 (3.16 to 3.24)	1	1
2	4.50 (4.45 to 4.55)	3.66 (3.61 to 3.70)	1.08 (1.06 to 1.10)	1.13 (1.11 to 1.15)
3	4.64 (4.58 to 4.49)	4.02 (3.97 to 4.07)	1.22 (1.20 to 1.24)	1.33 (1.31 to 1.36)
4	4.84 (4.79 to 4.90)	4.53 (4.48 to 4.59)	1.35 (1.33 to 1.38)	1.56 (1.54 to 1.60)
5	4.95 (4.88 to 5.02)	4.94 (4.87 to 5.01)	1.47 (1.44 to 1.50)	1.81 (1.77 to 1.85)

*Adjusted for other variables considered: age, deprivation and calendar year. Stratified by gender due to significant age-gender interaction.

IRR, incidence risk ratio; PYAR, person years at risk.

The incidence of type 2 diabetes by age was different for men and women (p value for interaction term <0.001). The risk of developing type 2 diabetes increased with age until the eighth decade for both men and women. In men, the incidence was 4.28 (95% CI 4.23 to 4.33) per 1000 PYAR in the 40–49 age band, with a peak incidence of 13.69 (95% CI 13.54 to 13.84) per 1000 PYAR between the ages of 70–79. The incidence in women was slightly lower than men between the ages of 40 and 49 at 3.16 (95% CI 3.12 to 3.21) per 1000 PYAR and peaked at a lower rate of 11.01 per 1000 PYAR (95% CI 10.89 to 11.13) between the ages of 70 and 79.

In 2009, the incidence per 1000 PYAR in men was 4.98 (95% CI 4.89 to 5.07), rising up to 5.06 per 1000 PYAR (95% CI 4.97 to 5.15) in 2013 (table 3). From 2014, the number of men newly diagnosed with type 2 diabetes markedly decreased to 3.56 per 1000 PYAR (95% CI 3.46 to 3.66) by 2018 figure 2). For women, in 2009 the incidence per 1000 PYAR was 4.40 (95% CI 4.32 to 4.48), peaking at 4.45 (95% CI 4.37 to 4.54) in 2013, before declining to 2.85 (2.76 to 2.93) per 1000 PYAR in 2018. The adjusted IRR for being diagnosed with type 2 diabetes was 0.68 (95% CI 0.66 to 0.70) for men in 2018 compared with 2013, and 0.62 (95% CI 0.60 to 0.65) for women in 2018 compared with 2013.

**Figure 2:**
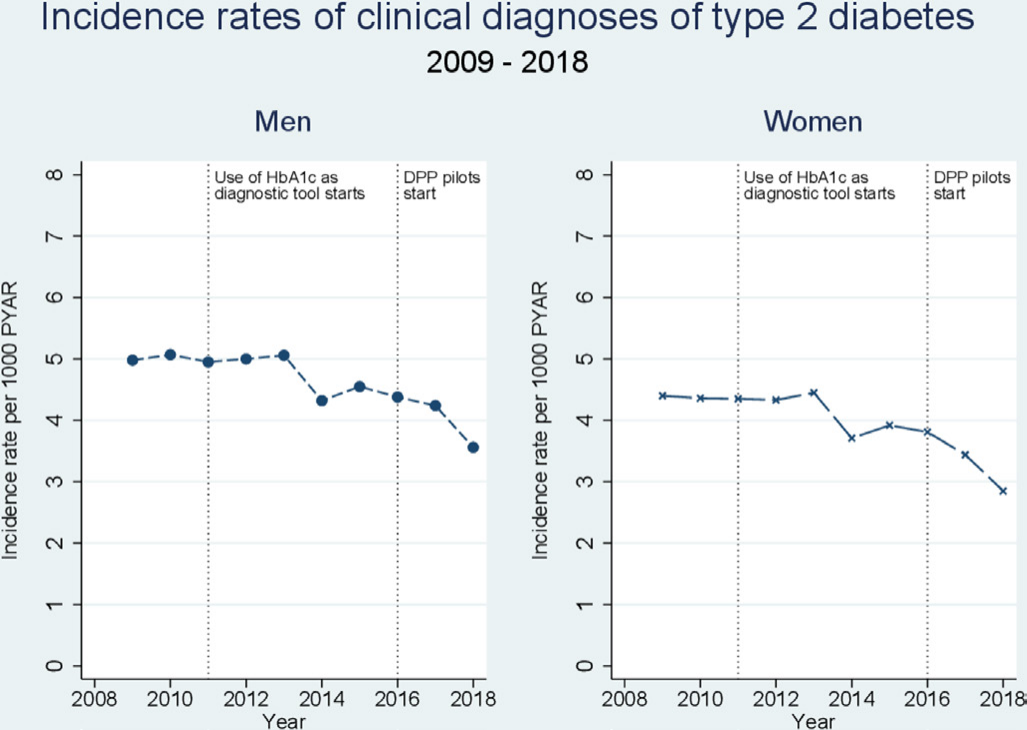
Incidence rates of clinical diagnosis of type 2 diabetes by calendar year 2009–2018. PYAR, person years at risk.

**Table 3:** Incidence of type 2 diabetes by calendar year

	Rate per 1000 PYAR (95% CI)		Adjusted IRR (95% CI)*	
	Men [annual change %]	Women [annual change %]	Men	Women
Year				
2009	4.98 (4.89 to 5.07)	4.40 (4.32 to 4.48)	0.98 (0.96 to 1.01)	0.98 (0.95 to 1.01)
2010	5.07 (4.98 to 5.17) [+1.81]	4.36 (4.28 to 4.45) [−0.91]	1.00 (0.98 to 1.03)	0.97 (0.95 to 1.00)
2011	4.95 (4.86 to 5.04) [−2.37]	4.35 (4.27 to 4.43) [−0.23]	0.98 (0.95 to 1.00)	0.98 (0.95 to 1.01)
2012	5.00 (4.91 to 5.09) [+1.01]	4.33 (4.24 to 4.41) [−0.46]	0.99 (0.96 to 1.01)	0.98 (0.95 to 1.00)
2013	5.06 (4.97 to 5.15) [+1.20]	4.45 (4.37 to 4.54) [+2.77]	1	1
2014	4.32 (4.23 to 4.40) [−14.62]	3.71 (3.63 to 3.79) [−16.63]	0.84 (0.82 to 0.87)	0.83 (0.81 to 0.86)
2015	4.55 (4.45 to 4.64) [+5.32]	3.92 (3.83 to 4.01) [+5.66]	0.88 (0.86 to 0.91)	0.88 (0.85 to 0.90)
2016	4.38 (4.28 to 4.48) [−3.74]	3.81 (3.72 to 3.91) [−2.81]	0.84 (0.82 to 0.87)	0.85 (0.82 to 0.87)
2017	4.24 (4.14 to 4.35) [−3.20]	3.44 (3.35 to 3.53) [−9.71]	0.81 (0.79 to 0.84)	0.75 (0.72 to 0.77)
2018	3.56 (3.46 to 3.66) [−16.04]	2.85 (2.76 to 2.93) [−17.15]	0.68 (0.66 to 0.70)	0.62 (0.60 to 0.65)

*Adjusted for other variables considered: age, deprivation and calendar year. Stratified by gender due to significant age-gender interaction.

IRR, incidence risk ratio; PYAR, person years at risk.

**Figure 3:**
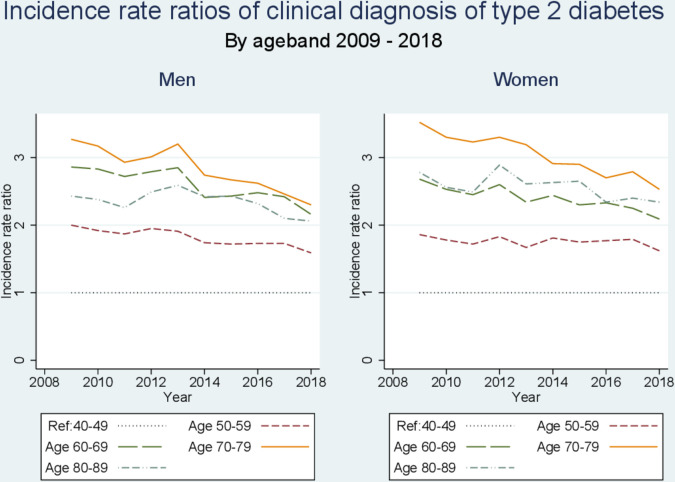
IRR for being diagnosed with type 2 diabetes in different age bands over time compared with age 40–49. IRR, incidence rate ratio.

The incidence rate ratios in older age groups, compared with the age band 40–49, declined after 2011 in both men and women (figure 3). There were significant drops in the incidence rates of the clinical diagnosis of type 2 diabetes in all age groups, with the largest decline seen in the 70–79 age band in men and women (online supplemental table 1).

The incidence of type 2 diabetes increased as deprivation increased, with an adjusted IRR of 1.47 (95% CI 1.44 to 1.50) for men in the most deprived quintile compared with the least deprived. The risk in women increased more with deprivation, with an IRR of 1.81 (95% CI 1.77 to 1.85) for women with the highest levels of deprivation compared with the least deprived. The incidence of diabetes was similar in men and women in the most deprived quintile.

### Incidence of pre-diabetes

Overall, men and women had similar risks of developing pre-diabetes (IRR for women compared with men: 1.01 95% CI 1.01 to 1.02) (table 4). The risk profile with age in pre-diabetes was similar to that seen in type 2 diabetes. The incidence of pre-diabetes increased with age, peaking in men in the 80–89 age band at 17.52 (95% CI 17.25 to 17.80) per 1000 PYAR and in the 70–79 ageband in women at 15.62 (95% CI 15.47 to 15.77) per 1000 PYAR.

**Table 4:** Incidence of pre-diabetes by age and deprivation

	Rate per 1000 PYAR (95% CI)		Adjusted IRR (95% CI)*	
	Men	Women	Men	Women
Overall	4.54 (4.52 to 4.52)	4.66 (4.64 to 4.68)	1	1.01 (1.01 to 1.02)
Age, years				
0–19	0.04 (0.04 to 0.05)	0.07 (0.06 to 0.07)	0.01 (0.01 to 0.02)	0.02 (0.02 to 0.02)
20–29	0.23 (0.21 to 0.24)	0.49 (0.47 to 0.51)	0.07 (0.07 to 0.07)	0.16 (0.15 to 0.17)
30–39	1.00 (0.97 to 1.02)	1.27 (1.24 to 1.31)	0.31 (0.30 to 0.32)	0.43 (0.42 to 0.44)
40–49	3.33 (3.28 to 3.38)	3.02 (2.97 to 3.06)	1	1
50–59	7.72 (7.64 to 7.80)	6.75 (6.68 to 6.82)	2.31 (2.26 to 2.36)	2.21 (2.17 to 2.26)
60–69	13.70 (13.58 to 13.83)	11.34 (11.23 to 11.45)	4.14 (4.06 to 4.22)	3.72 (3.64 to 3.79)
70–79	17.45 (17.27 to 17.63)	15.62 (15.47 to 15.77)	5.46 (5.35 to 5.57)	5.38 (5.27 to 5.49)
80–89	17.52 (17.25 to 17.80)	15.32 (15.12 to 15.51)	5.59 (5.45 to 5.72)	5.54 (5.41 to 5.66)
90–99	15.21 (14.54 to 15.91)	12.18 (11.82 to 12.54)	4.89 (4.65 to 5.14)	4.37 (4.22 to 4.54)
Townsend quintile				
1	4.48 (4.43 to 4.53)	4.16 (4.11 to 4.20)	1	1
2	4.65 (4.60 to 4.71)	4.45 (4.40 to 4.51)	1.05 (1.03 to 1.07)	1.08 (1.06 to 1.10)
3	4.66 (4.61 to 4.71)	4.82 (4.77 to 4.87)	1.13 (1.11 to 1.15)	1.22 (1.19 to 1.24)
4	4.43 (4.36 to 4.49)	4.99 (4.93 to 5.05)	1.19 (1.17 to 1.22)	1.36 (1.34 to 1.39)
5	4.47 (4.40 to 4.54)	5.28 (5.20 to 5.35)	1.26 (1.24 to 1.29)	1.52 (1.49 to 1.56)

*Adjusted for other variables considered: age, deprivation and calendar year. Stratified by gender due to significant age-gender interaction.

IRR, incidence risk ratio; PYAR, person years at risk.

**Figure 4:**
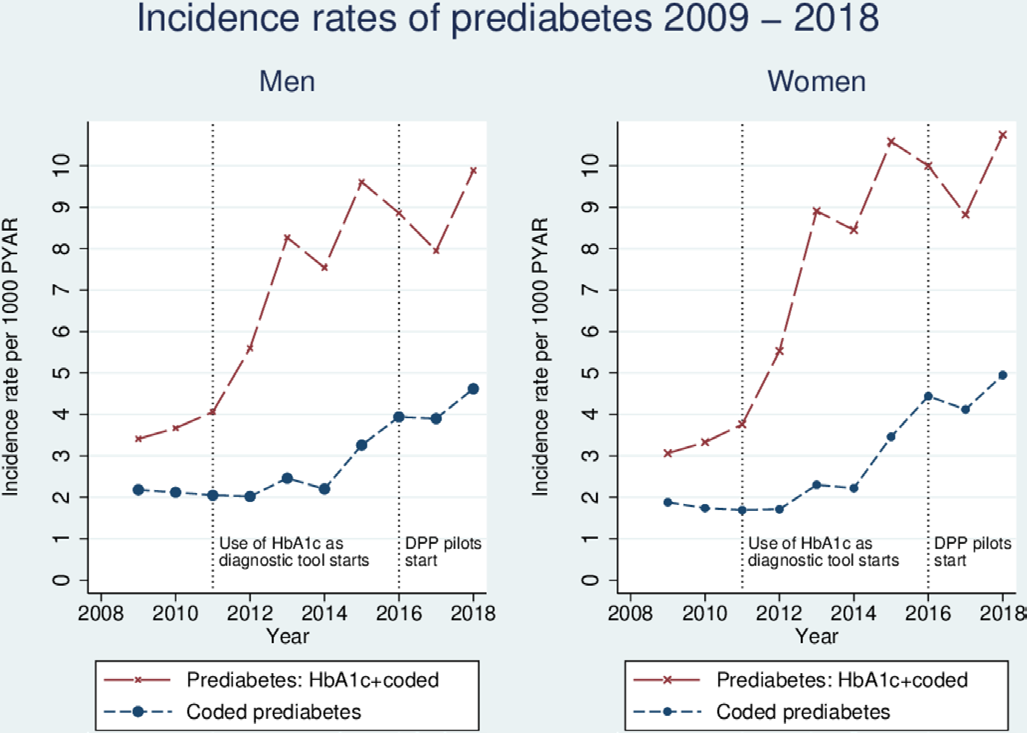
Incidence rates of diagnosis of pre-diabetes by calendar year 2009–2018. PYAR, person years at risk.

The incidence rates of people with pre-diabetes tripled by the end of the study period (table 5). In men, the incidence of pre-diabetes increased steadily from 3.41 per 1000 PYAR (95% CI 3.34 to 3.49) in 2009 to 9.89 per 1000 PYAR (95% CI 9.73 to 10.06) in 2018 (figure 4), with an adjusted IRR of 3.30 (95% CI 3.19 to 3.41) for 2018 compared with 2009. The incidence of pre-diabetes in women increased from 3.06 per 1000 PYAR (95% CI 2.99 to 3.13) to 10.75 per 1000 PYAR in 2018 (95% CI 10.58 to 10.93), an IRR of 4.16 (95% CI 4.03 to 4.30) in 2018 compared with 2009. The incident risk ratio for pre-diabetes rose steadily in the period 2013–2018, more than tripling in men and women. In this period, the IRR for type 2 diabetes dropped by a third in men and women (figure 2).

**Table 5:** Incidence of pre-diabetes by calendar year

	Rate per 1000 PYAR (95% CI)		Adjusted IRR (95% CI)*	
	Men [annual change %]	Women [annual change %]	Men	Women
Year				
2009	3.41 (3.34 to 3.49)	3.06 (2.99 to 3.13)	1	1
2010	3.67 (3.59 to 3.75) [+7.62]	3.33 (3.26 to 3.41) [+8.82]	1.07 (1.04 to 1.11)	1.08 (1.05 to 1.12)
2011	4.06 (3.98 to 4.15) [+10.63]	3.76 (3.68 to 3.84) [+12.91]	1.18 (1.14 to 1.22)	1.21 (1.17 to 1.26)
2012	5.60 (5.51 to 5.70) [+37.93]	5.53 (5.43 to 5.62) [+47.07]	1.59 (1.54 to 1.64)	1.71 (1.66 to 1.77)
2013	8.27 (8.15 to 8.39) [+47.68]	8.91 (8.79 to 9.04) [+61.12]	2.30 (2.23 to 2.37)	2.68 (2.60 to 2.77)
2014	7.54 (7.42 to 7.66) [−8.83]	8.45 (8.32 to 8.57) [−5.16]	2.21 (2.14 to 2.28)	2.75 (2.66 to 2.83)
2015	9.61 (9.46 to 9.75) [+27.45]	10.59 (10.45 to 10.74) [+25.33]	2.93 (2.84 to 3.02)	3.65 (3.54 to 3.76)
2016	8.86 (8.71 to 9.01) [−7.80]	10.00 (9.85 to 10.16) [−5.57]	2.85 (2.76 to 2.95)	3.69 (3.58 to 3.81)
2017	7.95 (7.80 to 8.10) [−10.27]	8.82 (8.67 to 8.97) [−11.80]	2.65 (2.57 to 2.74)	3.41 (3.30 to 3.53)
2018	9.89 (9.73 to 10.06) [+24.40]	10.75 (10.58 to 10.93) [+21.88]	3.30 (3.19 to 3.41)	4.16 (4.03 to 4.30)

*Adjusted for other variables considered: age, deprivation and calendar year. Stratified by gender due to significant age-gender interaction.

IRR, incidence risk ratio; PYAR, person years at risk.

The impact of deprivation on pre-diabetes risk was very similar to that seen in type 2 diabetes. The adjusted IRR in men was 1.26 (95% CI 1.24 to 1.29) in the highest quintile of deprivation compared with the lowest quintile. The risk of pre-diabetes in women increased by 52% in the most deprived quintile compared with the least deprived (IRR 1.52 95% CI 1.49 to 1.56).

Overall, pre-diabetes does not appear to be well coded in UK primary care records. Less than half of the records that fit the criteria for a diagnosis of non-diabetic hyperglycemia had an associated Read code (figure 4, online supplemental table 2).

## Discussion

The incidence of clinical diagnoses of type 2 diabetes recorded in GP electronic records dropped by 30% in men and women between 2009 and 2018. The risks of being clinically diagnosed with type 2 diabetes increased with deprivation and peaked in people between 70 and 79 years of age compared with those aged 40–49. While the recorded incidence of type 2 diabetes has dropped, rates of people with recorded pre-diabetes have risen steadily since 2011. Further, the risk of developing pre-diabetes increased with age and social deprivation, with women from the most deprived quintile having a 52% increase in the risk of developing pre-diabetes compared with women in the least deprived quintiles.

Two previous studies from the UK have confirmed evidence of increasing incidence of type 2 diabetes until 2010.^3 8^ Another study based on the UK Clinical Practice Research Datalink showed a drop in incidence between 2013 and 2014: in men, there was a drop from 51.26 to 42.59 per 10 000 patients, with a smaller drop in women from 35.98 to 31.83 per 10 000 patients.^27^ Internationally, studies from Portugal and Israel have demonstrated evidence of declines from 2011, with the incidence rate for developing type 2 diabetes in Portugal dropping from 6.49 per 1000 inhabitants in 2010–2012 to 6.30 in 2013–2015, and the incidence rate in Israel dropping from 13 per 1000 in 2011 to 10.8 in 2012.^28 29^ Recently published data from Denmark also showed a decrease in incidence of type 2 diabetes diagnosis between 2011 and 2014 around the time HbA1c was introduced as diagnostic tool, although the incidence rates increased again in the subsequent 2 years.^30^

A number of potential reasons have been postulated for reducing incidence, including diabetes prevention programs, public education, changing diet and the impact of screening.^7 31^ However, the Diabetes Prevention Programme was piloted in 2016, after the decrease trend in incidence in type 2 diabetes was observed in our data. There is also no evidence from NHS Digital data that trends in body weight have changed over this time period. The prevalence of overweight and obese adults in England has remained constant between 2009 and 2018, affecting more than 60% of men and 50% of women.^32^ Complications from type 2 diabetes take many years to develop, so any reductions in incidence will not lead to an immediate drop in prevalence rates as the condition is not immediately life-threatening.

Pre-diabetes has been associated with an increased risk of chronic kidney disease, cardiovascular disease and neuropathy,^33 34^ so the rising incidence of pre-diabetes has direct implications for health services. One of the challenges in interpreting changes in pre-diabetes diagnoses over time is the variation in the definitions of non-diabetic hyperglycemia.^35^ Pre-diabetes is a term commonly used by the American Diabetes Association (ADA) and is frequently used in the UK, while the WHO use ‘intermediate hyperglycemia’. They have different cut-offs for diagnosis based on fasting plasma glucose (5.6–6.9 mmol/L by the ADA, 6.1–6.9 mmol/L for WHO), and the ADA lowered the HbA1c threshold of diagnosis for pre-diabetes to 5.7% (39 mmol/mol) in 2010. In the UK, the National Institute for Health and Care Excellence (NICE) defines patients at high risk of developing type 2 diabetes using a fasting plasma glucose of 5.5–6.9 mmol/L or an HbA1c level of 6.0%–6.4% (42–47 mmol/mol). The NICE guidelines were published in 2012 and the thresholds did not change when reviewed in 2018.^15^ Based on blood samples provided for the Health Survey for England, the prevalence rate of pre-diabetes based on NICE guidance in a sampled population increased from 11.6% in 2003 to 35.3% in 2011 with an associated increase in mean population HbA1c.^17^

The UK does not have a formal population based screening program as current evidence does not suggest that this would be cost-effective.^36 37^ However, locally commissioned services and NHS health checks (started in April 2009) are opportunities where screening for diabetes can be routinely offered in primary care. While this activity does not seem to have increased the incidence rate of clinically diagnosed type 2 diabetes, it has resulted in large increases in the number of HbA1c results in the non-diabetic hyperglycemic range. The profile of people identified by HbA1c is different to diagnostic tests based on blood glucose sampling and there have been suggestions this may lead to underdiagnosis of type 2 diabetes when using HbA1c as a sole diagnostic test.^38–40^ This could be one possible explanation why the large increase in abnormal HbA1c results and diagnoses of pre-diabetes has not been accompanied by an increased rate of clinically diagnosed type 2 diabetes. Clinical diagnosis rates for pre-diabetes may continue to rise as GP-recorded prevalence rates of non-diabetic hyperglycemia in England 2018–2019 were less than 5% in the National Diabetes Audit^41^ and our results suggest that most non-diabetic hyperglycemia is currently not being coded as pre-diabetes.

When examining the age-specific incidence rates for type 2 diabetes, it was revealed that clinical diagnosis rates are dropping fastest in older adults aged 60 and over. A recent study in middle-aged and older Chinese patients found that the current HbA1c threshold had a low sensitivity of just 35.6%, possibly due to lower red cell counts in older people.^42^ There are also known ethnic variations in HbA1c and comparisons with an oral glucose tolerance test showed a lower sensitivity when using current HbA1c cut-offs for detecting diabetes in ethnic minority groups in the USA.^43^ The current single absolute cut-off for HbA1c to diagnose diabetes may have significant limitations as older adults and ethnic minority groups are populations at high risk of developing type 2 diabetes. If the current diagnostic test lacks sensitivity and delays diagnosis in certain high risk groups, this could lead to delays in accessing treatment and an increasing risk of developing complications. To mitigate this, cardiovascular risk factors may need to be managed as actively in pre-diabetes as they are in type 2 diabetes. This approach would be supported by recent evidence showing people with blood glucose levels just above the threshold of diagnosis of type 2 diabetes have improved mortality compared with those just below.^44^

This study has a number of strengths. It includes data from nearly half a million people with type 2 diabetes and follow-up data over 10 years and IMRD data have been shown to be broadly representative of the UK population, GPs are incentivized to keep up to date registers for diabetes^45^ and most routine care for type 2 diabetes in the UK happens in primary care.^46^ The main limitation of this study comes from the use of routinely recorded primary care data, which would not capture diabetes and pre-diabetes cases missed by GPs, and it does not include people with type 2 diabetes without a GP. The definition for pre-diabetes was based on Read codes for impaired glucose tolerance, impaired fasting glucose tolerance and pre-diabetes or HbA1c levels based on NICE definitions of people at high risk of developing type 2 diabetes, so these results may not be directly comparable to countries using different diagnostic criteria for non-diabetic hyperglycemia. Although there is no national system for maintaining pre-diabetes registers, there are often local enhanced schemes to incentivize maintenance of pre-diabetes registers, so they are likely to be well maintained. Some of the increase in rates of pre-diabetes diagnoses will reflect this increased activity from local incentive schemes and the roll out of the National Diabetes Prevention Programme. However, the trend in increasing rates of diagnosis of pre-diabetes with a steady decline in the clinical diagnosis of type 2 diabetes prior to diabetes prevention programs being widely available raises important questions about the sensitivity and specificity of HbA1c as a diagnostic test in type 2 diabetes compared with blood glucose based diagnostic tests. As the data for this study were collected from routine clinical practice, data quality for some characteristics like body mass index and ethnicity was variable, so the reporting on these was limited. However, a previous study has described differences in the prevalence of type 2 diagnoses in a similar dataset, with adjusted ORs for the prevalence of diagnoses of type 2 diabetes being 2.36 (95% CI 2.26 to 2.47) in Asian patients and 1.65 (95% CI 1.56 to 1.73) in Black patients, compared with White patients.^47^

Further research is needed to understand why relative rates of clinical diagnosis of type 2 diabetes in the UK appear to be falling in people over 60. We also need to be able to risk stratify the increasing numbers of people with pre-diabetes as it is possible that the current absolute threshold for HbA1c is not sensitive enough for some patient groups and we may be delaying or missing a diagnosis of type 2 diabetes.

## Conclusion

The incidence rate of new clinical diagnoses of type 2 diabetes recorded in primary care records in the UK has dropped by a third since 2013, while the rates of pre-diabetes have tripled. More people in the UK are now being diagnosed with pre-diabetes than type 2 diabetes. The steepest decline in clinical diagnoses of type 2 diabetes was in people aged 60–79 years old and the changes accelerated a few years after the introduction of HbA1c as a diagnostic test for type 2 diabetes. Further research is needed to understand if the current single threshold for HbA1c used in diagnosing type 2 diabetes is appropriate in all age groups and to understand the risks for the increasing number of people fitting the diagnostic criteria for pre-diabetes.

## Data Availability

Data are available on reasonable request. Data have been extracted from pseudonymised routinely collected UK primary care records from the IQVIA Medical Research-UK data.

## References

[1] Cho NH, Shaw JE, Karuranga S, et al. IDF diabetes atlas: global estimates of diabetes prevalence for 2017 and projections for 2045. Diabetes Res Clin Pract 2018;138:271–81. 10.1016/j.diabres.2018.02.023 29496507

[2] Menke A, Casagrande S, Geiss L, et al. Prevalence of and trends in diabetes among adults in the United States, 1988-2012. JAMA 2015;314:1021–9. 10.1001/jama.2015.10029 26348752

[3] Sharma M, Nazareth I, Petersen I. Trends in incidence, prevalence and prescribing in type 2 diabetes mellitus between 2000 and 2013 in primary care: a retrospective cohort study. BMJ Open 2016;6:e010210. 10.1136/bmjopen-2015-010210 PMC4735176 26769791

[4] National Collaborating Centre for Chronic Conditions. Type 2 diabetes national clinical guideline for management in primary and secondary care (update). London: Royal College of Physicians, 2008. 21678628

[5] Diabetes UK. DIABETES: FACTS AND STATS [Internet]. Diabetes UK, 2014. Available: http://www.diabetes.org.uk/Documents/About%20Us/Statistics/Diabetes-key-stats-guidelines-April2014.pdf

[6] Health & Social Care Information Centre. Quality and Outcomes Framework - Prevalence, Achievements and Exceptions Report, 2014. Available: http://www.hscic.gov.uk/catalogue/PUB15751/qof-1314-report-V1.1.pdf

[7] Magliano DJ, Islam RM, Barr ELM, et al. Trends in incidence of total or type 2 diabetes: systematic review. BMJ 2019;366:l5003. 31511236 10.1136/bmj.l5003 PMC6737490

[8] Holden SH, Barnett AH, Peters JR, et al. The incidence of type 2 diabetes in the United Kingdom from 1991 to 2010. Diabetes Obes Metab 2013;15:844–52. 10.1111/dom.12123 23675742

[9] Buysschaert M, Bergman M. Definition of prediabetes. Med Clin North Am 2011;95:289–97. 10.1016/j.mcna.2010.11.002 21281833

[10] Tabák AG, Herder C, Rathmann W, et al. Prediabetes: a high-risk state for diabetes development. Lancet 2012;379:2279–90. 10.1016/S0140-6736(12)60283-9 22683128 PMC3891203

[11] Plantinga LC, Crews DC, Coresh J, et al. Prevalence of chronic kidney disease in US adults with undiagnosed diabetes or prediabetes. Clin J Am Soc Nephrol 2010;5:673–82. 10.2215/CJN.07891109 20338960 PMC2849697

[12] Thomas G, Sehgal AR, Kashyap SR, et al. Metabolic syndrome and kidney disease: a systematic review and meta-analysis. Clin J Am Soc Nephrol 2011;6:2364–73. 10.2215/CJN.02180311 21852664 PMC3186450

[13] Tesfaye S, Boulton AJM, Dyck PJ, et al. Diabetic neuropathies: update on definitions, diagnostic criteria, estimation of severity, and treatments. Diabetes Care 2010;33:2285–93. 10.2337/dc10-1303 20876709 PMC2945176

[14] American Diabetes Association. Diagnosis and classification of diabetes mellitus. Diabetes Care 2014;37(Suppl 1):S81–90. 10.2337/dc14-S081 24357215

[15] National Institute for Health and Care Excellence. Recommendations | Type 2 diabetes: prevention in people at high risk | Guidance. Available: https://www.nice.org.uk/guidance/PH38/chapter/Recommendations#risk-assessment [Accessed 26 Jun 2020].

[16] Cosentino F, Grant PJ, Aboyans V, et al. 2019 ESC guidelines on diabetes, pre-diabetes, and cardiovascular diseases developed in collaboration with the EASD: the task force for diabetes, pre-diabetes, and cardiovascular diseases of the European Society of cardiology (ESC) and the European association for the study of diabetes (EASD). European heart Journal 2020;41:255–323. 10.1093/eurheartj/ehz486 31497854

[17] Mainous AG, Tanner RJ, Baker R, et al. Prevalence of prediabetes in England from 2003 to 2011: population-based, cross-sectional study. BMJ Open 2014;4:e005002. 10.1136/bmjopen-2014-005002 PMC4054625 24913327

[18] Khan NF, Harrison SE, Rose PW. Validity of diagnostic coding within the general practice research database: a systematic review. Br J Gen Pract 2010;60:e128–36. 10.3399/bjgp10X483562 20202356 PMC2828861

[19] Blak BT, Thompson M, Dattani H, et al. Generalisability of the health improvement network (thin) database: demographics, chronic disease prevalence and mortality rates. Inform Prim Care 2011;19:251–5. 10.14236/jhi.v19i4.820 22828580

[20] Martín-Merino E, Fortuny J, Rivero E, et al. Validation of diabetic retinopathy and maculopathy diagnoses recorded in a U.K. primary care database. Diabetes Care 2012;35:762–7. 10.2337/dc11-2069 22357184 PMC3308315

[21] Chisholm J. The read clinical classification. BMJ 1990;300:1092. 10.1136/bmj.300.6732.1092 2344534 PMC1662793

[22] Hardoon S, Hayes JF, Blackburn R, et al. Recording of severe mental illness in United Kingdom primary care, 2000-2010. PLoS One 2013;8:e82365. 10.1371/journal.pone.0082365 24349267 PMC3861391

[23] PLOS Medicine Editors. Observational studies: getting clear about transparency. PLoS Med 2014;11:e1001711. 10.1371/journal.pmed.1001711 25158064 PMC4144975

[24] von Elm E, Altman DG, Egger M, et al. Strengthening the reporting of observational studies in epidemiology (STROBE) statement: guidelines for reporting observational studies. BMJ 2007;335:806–8. 10.1136/bmj.39335.541782.AD 17947786 PMC2034723

[25] Horsfall L, Walters K, Petersen I. Identifying periods of acceptable computer usage in primary care research databases. Pharmacoepidemiol Drug Saf 2013;22:64–9. 10.1002/pds.3368 23124958

[26] Maguire A, Blak BT, Thompson M. The importance of defining periods of complete mortality reporting for research using automated data from primary care. Pharmacoepidemiol Drug Saf 2009;18:76–83. 10.1002/pds.1688 19065600

[27] Zghebi SS, Steinke DT, Carr MJ, et al. Examining trends in type 2 diabetes incidence, prevalence and mortality in the UK between 2004 and 2014. Diabetes Obes Metab 2017;19:1537–45. 10.1111/dom.12964 28387052

[28] de Sousa-Uva M, Antunes L, Nunes B, et al. Trends in diabetes incidence from 1992 to 2015 and projections for 2024: a Portuguese general practitioner's network study. Prim Care Diabetes 2016;10:329–33. 10.1016/j.pcd.2016.05.003 27363730

[29] Karpati T, Cohen-Stavi CJ, Leibowitz M, et al. Towards a subsiding diabetes epidemic: trends from a large population-based study in Israel. Popul Health Metr 2014;12:1–8. 10.1186/s12963-014-0032-y 25400512 PMC4233034

[30] Carstensen B, Rønn PF, Jørgensen ME. Prevalence, incidence and mortality of type 1 and type 2 diabetes in Denmark 1996–2016. BMJ Open Diabetes Res Care 2020;8:e001071. 10.1136/bmjdrc-2019-001071 PMC7265004 32475839

[31] Lean M, McCombie L, McSorely J. Trends in type 2 diabetes. BMJ 2019;366:l5407. 10.1136/bmj.l5407 31511237

[32] NHS Digital. Statistics on obesity, physical activity and diet, England, 2019. Available: https://digital.nhs.uk/data-and-information/publications/statistical/statistics-on-obesity-physical-activity-and-diet/statistics-on-obesity-physical-activity-and-diet-england-2019 [Accessed 17 Jun 2020].

[33] Huang Y, Cai X, Mai W, et al. Association between prediabetes and risk of cardiovascular disease and all cause mortality: systematic review and meta-analysis. BMJ 2016:i5953. 10.1136/bmj.i5953 27881363 PMC5121106

[34] Echouffo-Tcheugui JB, Narayan KM, Weisman D, et al. Association between prediabetes and risk of chronic kidney disease: a systematic review and meta-analysis. Diabet Med 2016;33:1615–24. 10.1111/dme.13113 26997583

[35] Hostalek U. Global epidemiology of prediabetes - present and future perspectives. Clin Diabetes Endocrinol 2019;5:5. 10.1186/s40842-019-0080-0 31086677 PMC6507173

[36] Waugh NR, Shyangdan D, Taylor-Phillips S, et al. Screening for type 2 diabetes: a short report for the National screening Committee. Health Technol Assess 2013;17:1–90. 10.3310/hta17350 PMC4780946 23972041

[37] Sargeant LA, Simmons RK, Barling RS, et al. Who attends a UK diabetes screening programme? findings from the ADDITION-Cambridge study. Diabet Med 2010;27:995–1003. 10.1111/j.1464-5491.2010.03056.x 20722672 PMC3428846

[38] Malkani S, Mordes JP. Implications of using hemoglobin A1c for diagnosing diabetes mellitus. Am J Med 2011;124:395–401. 10.1016/j.amjmed.2010.11.025 21531226 PMC3086708

[39] Diabetes Care. Screening for diabetes and pre-diabetes with proposed A1C-Based diagnostic criteria. Available: https://care.diabetesjournals.org/content/33/10/2184.long [Accessed 6 May 2020]. 10.2337/dc10-0433 PMC2945158 20639452

[40] Wiley Online Library. HbA1c in diagnosing and predicting Type 2 diabetes in impaired glucose tolerance: the Finnish Diabetes Prevention Study - Pajunen - 2011 - Diabetic Medicine [Accessed 6 May 2020]. 10.1111/j.1464-5491.2010.03183.x 21166843

[41] NHS Digital. National diabetes audit non-diabetic hyperglycaemia, 2018- 2019, diabetes prevention programme. Available: https://digital.nhs.uk/data-and-information/publications/statistical/national-diabetes-audit/non-diabetic-hyperglycamia-2018-2019-diabetes-prevention-programme [Accessed 30 Nov 2020].

[42] Wu L, Lin H, Gao J, et al. Effect of age on the diagnostic efficiency of HbA1c for diabetes in a Chinese middle-aged and elderly population: the Shanghai Changfeng study. PLoS One 2017;12:e0184607. 10.1371/journal.pone.0184607 28886160 PMC5591004

[43] Araneta MRG, Grandinetti A, Chang HK. A1C and diabetes diagnosis among Filipino Americans, Japanese Americans, and native Hawaiians. Diabetes Care 2010;33:2626–8. 20833866 10.2337/dc10-0958 PMC2992202

[44] Petersen I, Nicolaisen SK, Ricciardi F, et al. Impact of being eligible for type 2 diabetes treatment on all-cause mortality and cardiovascular events: regression discontinuity design study. Clin Epidemiol 2020;12:569–77. 10.2147/CLEP.S251704 32606982 PMC7294562

[45] NHS Digital. Quality and Outcomes Framework, Achievement, prevalence and exceptions data - 2017-18 [PAS]. Available: https://digital.nhs.uk/data-and-information/publications/statistical/quality-and-outcomes-framework-achievement-prevalence-and-exceptions-data/2017-18 [Accessed 11 Aug 2020].

[46] Rushforth B, McCrorie C, Glidewell L, et al. Barriers to effective management of type 2 diabetes in primary care: qualitative systematic review. Br J Gen Pract 2016;66:e114–27. 10.3399/bjgp16X683509 26823263 PMC4723210

[47] Pham TM, Carpenter JR, Morris TP, et al. Ethnic differences in the prevalence of type 2 diabetes diagnoses in the UK: cross-sectional analysis of the health improvement network primary care database. Clin Epidemiol 2019;11:1081–8. 10.2147/CLEP.S227621 32021464 PMC6948201

