# Supplementary material for: Time trends in the incidence of clinically diagnosed type 2 diabetes and pre-diabetes in the UK 2009–2018: a retrospective cohort study
Source: BMJ Open Diabetes Res Care. 2021 Mar 19;9(1):e001989. doi: 10.1136/bmjdrc-2020-001989 (PMC7986873; doi:10.1136/bmjdrc-2020-001989)
Supplement: Supplementary data [file bmjdrc-2020-001989supp001.pdf]

Appendix 1: Read code lists

Diabetes

medcode	description
13AB.00	Diabetic lipid lowering diet
13AC.00	Diabetic weight reducing diet
13B1.00	Diabetic diet
1434.00	H/O: diabetes mellitus
14F4.00	H/O: Admission in last year for diabetes foot problem
1M8..00	Diabetic peripheral neuropathic pain
2BBF.00	Retinal abnormality - diabetes related
2BBJ.00	O/E - no right diabetic retinopathy
2BBK.00	O/E - no left diabetic retinopathy
2BBL.00	O/E - diabetic maculopathy present both eyes
2BBM.00	O/E - diabetic maculopathy absent both eyes
2BBP.00	O/E - right eye background diabetic retinopathy
2BBQ.00	O/E - left eye background diabetic retinopathy
2BBR.00	O/E - right eye preproliferative diabetic retinopathy
2BBS.00	O/E - left eye preproliferative diabetic retinopathy
2BBT.00	O/E - right eye proliferative diabetic retinopathy
2BBV.00	O/E - left eye proliferative diabetic retinopathy
2BBW.00	O/E - right eye diabetic maculopathy
2BBX.00	O/E - left eye diabetic maculopathy
2BBk.00	O/E - right eye stable treated prolif diabetic retino
2BBl.00	O/E - left eye stable treated prolif diabetic retinop
2BBo.00	O/E - sight threatening diabetic retinopathy
2BBr.00	Impair vision due diab retinop
2G51000	Foot abnormality - diabetes related
2G5A.00	O/E - Right diabetic foot at risk
2G5B.00	O/E - Left diabetic foot at risk
2G5C.00	Foot abnormality - diabetes related
2G5E.00	O/E - Right diabetic foot at low risk
2G5F.00	O/E - Right diabetic foot at moderate risk
2G5G.00	O/E - Right diabetic foot at high risk
2G5H.00	O/E - Right diabetic foot - ulcerated
2G5I.00	O/E - Left diabetic foot at low risk
2G5J.00	O/E - Left diabetic foot at moderate risk
2G5K.00	O/E - Left diabetic foot at high risk
2G5L.00	O/E - Left diabetic foot - ulcerated
2G5V.00	O/E - right chronic diabetic foot ulcer
2G5W.00	O/E - left chronic diabetic foot ulcer
2G5d.00	O/E - Left diabetic foot at increased risk
2G5e.00	O/E - Right diabetic foot at increased risk
3882.00	Diabetes well being questionnaire
3883.00	Diabetes treatment satisfaction questionnaire
42c1.00	HbA1 7 - 10% - borderline control
42c2.00	HbA1 > 10% - bad control
661M400	Diabetes self-management plan agreed
661N400	Diabetes self-management plan review
66A..00	Diabetic monitoring
66A1.00	Initial diabetic assessment

Pal K, *et al. BMJ Open Diab Res Care* 2021; 9:e001989. doi: 10.1136/bmjdrc-2020-001989

66A2.00	Follow-up diabetic assessment
66A3.00	Diabetic on diet only
66A4.00	Diabetic on oral treatment
66A5.00	Diabetic on insulin
66A8.00	Has seen dietician - diabetes
66A9.00	Understands diet - diabetes
66AA.11	Injection sites - diabetic
66AD.00	Fundoscopy - diabetic check
66AG.00	Diabetic drug side effects
66AH.00	Diabetic treatment changed
66AH000	Conversion to insulin
66AH100	Conversion to insulin in secondary care
66AH300	Conversion to non-insulin injectable medication
66AI.00	Diabetic - good control
66AJ.00	Diabetic - poor control
66AJ.11	Unstable diabetes
66AJ100	Brittle diabetes
66AJz00	Diabetic - poor control NOS
66AK.00	Diabetic - cooperative patient
66AL.00	Diabetic-uncooperative patient
66AM.00	Diabetic - follow-up default
66AN.00	Date diabetic treatment start
66AO.00	Date diabetic treatment stopp.
66AP.00	Diabetes: practice programme
66AQ.00	Diabetes: shared care programme
66AQ000	Unsuitable for diabetes year of care programme
66AQ100	Declined consent for diabetes year of care programme
66AR.00	Diabetes management plan given
66AS.00	Diabetic annual review
66AT.00	Annual diabetic blood test
66AU.00	Diabetes care by hospital only
66AV.00	Diabetic on insulin and oral treatment
66AW.00	Diabetic foot risk assessment
66AX.00	Diabetes: shared care in pregnancy - diabetol and obs
66AY.00	Diabetic diet - good compliance
66AZ.00	Diabetic monitoring NOS
66Aa.00	Diabetic diet - poor compliance
66Ab.00	Diabetic foot examination
66Ac.00	Diabetic peripheral neuropathy screening
66Ai.00	Diabetic 6 month review
66Ak.00	Diabetic monitoring - lower risk albumin excretion
66Al.00	Diabetic monitoring - higher risk albumin excretion
66Am.00	Insulin dose changed
66Ao.00	Diabetes type 2 review
66Ap.00	Insulin treatment initiated
66Aq.00	Diabetic foot screen
66Ar.00	Insulin treatment stopped
66As.00	Diabetic on subcutaneous treatment
66At.00	Diabetic dietary review
66At100	Type II diabetic dietary review
66At111	Type 2 diabetic dietary review

66Au.00 Diabetic erectile dysfunction review
66Av.00 Diabetic assessment of erectile dysfunction
66Az.00 High risk of diabetes mellitus annual review
66o5.00 Diabetic on oral treatment and glucagon-like peptide 1
66o6.00 Diabetic on insulin and glucagon-like peptide 1
6761.00 Diabetic pre-pregnancy counselling
679L000 Education in self management of diabetes
679R.00 Patient offered diabetes structured education program
67D8.00 Provision of diabetes clinical summary
67IJ100 Pre-conception advice for diabetes mellitus
68A7.00 Diabetic retinopathy screening
68A9.00 Diabetic retinopathy screening offered
68AB.00 Diabetic digital retinopathy screening offered
889A.00 Diab mellit insulin-glucose infus acute myocardial in
8A12.00 Diabetic crisis monitoring
8A13.00 Diabetic stabilisation
8B3l.00 Diabetes medication review
8BAi.00 Insulin passport completed
8BAj.00 Informed dissent not to carry insulin passport
8BAm.00 Insulin passport checked
8BAp.00 Insulin passport not checked
8BL2.00 Patient on maximal tolerated therapy for diabetes
8CA4100 Pt advised re diabetic diet
8CE0100 Insulin alert patient information booklet given
8CE0200 Insulin passport given
8CMW700 Diabetes clinical pathway
8CP2.00 Transition of diabetes care options discussed
8CR2.00 Diabetes clinical management plan
8CS0.00 Diabetes care plan agreed
8H2J.00 Admit diabetic emergency
8H3O.00 Non-urgent diabetic admission
8H4F.00 Referral to diabetologist
8H4e.00 Referral to diabetes special interest general practit
8H7C.00 Refer, diabetic liaison nurse
8H7f.00 Referral to diabetes nurse
8H7r.00 Refer to diabetic foot screener
8HBG.00 Diabetic retinopathy 12 month review
8HBH.00 Diabetic retinopathy 6 month review
8HHy.00 Referral to diabetic register
8HKE.00 Diabetology D.V. requested
8HLE.00 Diabetology D.V. done
8HME.00 Listed for Diabetology admissn
8HTE100 Referral to community diabetes clinic
8HTe.00 Referral to diabetes preconception counselling clinic
8HTi.00 Referral to multidisciplinary diabetic clinic
8HTk.00 Referral to diabetic eye clinic
8HVU.00 Private referral to diabetologist
8Hg4.00 Discharged from care of diabetes specialist nurse
8HgC.00 Discharged from diabetes shared care programme
8Hj1.00 Family/carer referral to diabetes structured education
8Hj4.00 Referral to DESMOND diabetes structured education programme

8Hl1.00 Referral for diabetic retinopathy screening
8Hl4.00 Referral to community diabetes specialist nurse
8Hlc.00 Referral to community diabetes service
8I3W.00 Diabetic foot examination declined
8I3X.00 Diabetic retinopathy screening refused
8I57.00 Patient held diabetic record declined
8I6F.00 Diabetic retinopathy screening not indicated
8I6G.00 Diabetic foot examination not indicated
8I83.00 Did not complete DESMOND diabetes structured educat program
8IAs.00 Diabetic dietary review declined
8IE2.00 Diabetes care plan declined
8IEQ.00 Referral to community diabetes specialist nurse decli
918T.00 Diabetes key contact
9360.00 Patient held diabetic record issued
93C4.00 Patient consent given for addition to diabetic regist
9M00.00 Informed consent for diabetes national audit
9M10.00 Informed dissent for diabetes national audit
9N0m.00 Seen in diabetic nurse consultant clinic
9N0n.00 Seen in community diabetes specialist clinic
9N0o.00 Seen in community diabetic specialist nurse clinic
9N1Q.00 Seen in diabetic clinic
9N1i.00 Seen in diabetic foot clinic
9N1o.00 Seen in multidisciplinary diabetic clinic
9N1v.00 Seen in diabetic eye clinic
9N2d.00 Seen by diabetologist
9N2i.00 Seen by diabetic liaison nurse
9N4I.00 DNA - Did not attend diabetic clinic
9N4p.00 Did not attend diabetic retinopathy clinic
9NJy.00 In-house diabetic foot screening
9NM0.00 Attending diabetes clinic
9NN8.00 Under care of diabetologist
9NN9.00 Under care of diabetes specialist nurse
9NN9000 Under care hos diab spec nurse
9NN9100 Under care com diab spec nurse
9NND.00 Under care of diabetic foot screener
9NiD.00 Did not attend DESMOND diabetes structured education program
9NiZ.00 Did not attend diabetes foot screening
9Nl4.00 Seen by general practitioner special interest in diab
9NlP100 Seen by diabetes speclst nurse
9OL..00 Diabetes monitoring admin.
9OL..11 Diabetes clinic administration
9OL1.00 Attends diabetes monitoring
9OL2.00 Refuses diabetes monitoring
9OL3.00 Diabetes monitoring default
9OL4.00 Diabetes monitoring 1st letter
9OL5.00 Diabetes monitoring 2nd letter
9OL6.00 Diabetes monitoring 3rd letter
9OL7.00 Diabetes monitor.verbal invite
9OL8.00 Diabetes monitor.phone invite
9OL9.00 Diabetes monitoring deleted
9OLA.00 Diabetes monitor. check done

9OLA.11	Diabetes monitored
9OLD.00	Diabetic patient unsuitable for digital retinal photo
9OLJ.00	DAFNE diabetes structured education programme complet
9OLK.00	DESMOND diabetes structured education programme completed
9OLN.00	Diabetes monitor invitation by SMS (short message ser
9OLZ.00	Diabetes monitoring admin.NOS
9Oy..00	Diabetes screening administration
9b92000	Diabetic medicine
9h4..00	Exception reporting: diabetes quality indicators
9h41.00	Excepted from diabetes qual indicators: Patient unsui
9h42.00	Excepted from diabetes quality indicators: Informed d
9h43.00	Excepted from diabetes qual indicators: service unava
9m0..00	Diabetic retinopathy screening administrative status
9m00.00	Eligible for diabetic retinopathy screening
9m01.00	Ineligible for diabetic retinopathy screening
9m02.00	Eligb temp inactv diab ret scr
9m03.00	Eligb perm inactv diab ret scr
9m04.00	Excluded from diabetic retinopathy screening
9m05.00	Excluded from diabetic retinopathy screening as moved
9m06.00	Excluded from diabetic retinopathy screening as decea
9m07.00	Excluded diabetc retinop screen as under care ophthal
9m08.00	Exclu diab ret screen as blind
9m0A.00	Declined diabetic retinop scrn
9m0B.00	Ex diab ret scr no cntct detls
9m0C.00	Excluded frm diabetic retinopathy screen as terminal
9m0D.00	Excluded from diabetic retinopthy screen as learn dis
9m0E.00	Excluded from diabetic retinopathy screen physical di
C10..00	Diabetes mellitus
C100.00	Diabetes mellitus with no mention of complication
C100000	Diabetes mellitus, juvenile type, no mention of compl
C100011	Insulin dependent diabetes mellitus
C100100	Diabetes mellitus, adult onset, no mention of complic
C100111	Maturity onset diabetes
C100112	Non-insulin dependent diabetes mellitus
C100z00	Diabetes mellitus NOS with no mention of complication
C101.00	Diabetes mellitus with ketoacidosis
C101000	Diabetes mellitus, juvenile type, with ketoacidosis
C101100	Diabetes mellitus, adult onset, with ketoacidosis
C101y00	Other specified diabetes mellitus with ketoacidosis
C101z00	Diabetes mellitus NOS with ketoacidosis
C102.00	Diabetes mellitus with hyperosmolar coma
C102100	Diabetes mellitus, adult onset, with hyperosmolar com
C102z00	Diabetes mellitus NOS with hyperosmolar coma
C103.00	Diabetes mellitus with ketoacidotic coma
C103100	Diabetes mellitus, adult onset, with ketoacidotic com
C103y00	Other specified diabetes mellitus with coma
C103z00	Diabetes mellitus NOS with ketoacidotic coma
C104.00	Diabetes mellitus with renal manifestation
C104.11	Diabetic nephropathy
C104100	Diabetes mellitus, adult onset, with renal manifestat
C104y00	Other specified diabetes mellitus with renal complica

Pal K, et al. BMJ Open Diab Res Care 2021; 9:e001989. doi: 10.1136/bmjdrc-2020-001989

C104z00	Diabetes mellitus with nephropathy NOS
C105.00	Diabetes mellitus with ophthalmic manifestation
C105000	Diabetes mellitus, juvenile type, + ophthalmic manife
C105100	Diabetes mellitus, adult onset, + ophthalmic manifest
C105y00	Other specified diabetes mellitus with ophthalmic com
C105z00	Diabetes mellitus NOS with ophthalmic manifestation
C106.00	Diabetes mellitus with neurological manifestation
C106.11	Diabetic amyotrophy
C106.12	Diabetes mellitus with neuropathy
C106.13	Diabetes mellitus with polyneuropathy
C106000	Diabetes mellitus, juvenile, + neurological manifesta
C106100	Diabetes mellitus, adult onset, + neurological manife
C106y00	Other specified diabetes mellitus with neurological c
C106z00	Diabetes mellitus NOS with neurological manifestation
C107.00	Diabetes mellitus with peripheral circulatory disorde
C107.11	Diabetes mellitus with gangrene
C107.12	Diabetes with gangrene
C107000	Diabetes mellitus, juvenile +peripheral circulatory d
C107100	Diabetes mellitus, adult, + peripheral circulatory di
C107200	Diabetes mellitus, adult with gangrene
C107400	NIDDM with peripheral circulatory disorder
C107y00	Other specified diabetes mellitus with periph circ co
C107z00	Diabetes mellitus NOS with peripheral circulatory dis
C109.00	Non-insulin dependent diabetes mellitus
C109.11	NIDDM - Non-insulin dependent diabetes mellitus
C109.12	Type 2 diabetes mellitus
C109.13	Type II diabetes mellitus
C109000	Non-insulin-dependent diabetes mellitus with renal co
C109011	Type II diabetes mellitus with renal complications
C109012	Type 2 diabetes mellitus with renal complications
C109100	Non-insulin-dependent diabetes mellitus with ophthalm
C109111	Type II diabetes mellitus with ophthalmic complicatio
C109112	Type 2 diabetes mellitus with ophthalmic complication
C109200	Non-insulin-dependent diabetes mellitus with neuro co
C109211	Type II diabetes mellitus with neurological complicat
C109212	Type 2 diabetes mellitus with neurological complicati
C109300	Non-insulin-dependent diabetes mellitus with multiple
C109311	Type II diabetes mellitus with multiple complications
C109312	Type 2 diabetes mellitus with multiple complications
C109400	Non-insulin dependent diabetes mellitus with ulcer
C109411	Type II diabetes mellitus with ulcer
C109412	Type 2 diabetes mellitus with ulcer
C109500	Non-insulin dependent diabetes mellitus with gangrene
C109511	Type II diabetes mellitus with gangrene
C109512	Type 2 diabetes mellitus with gangrene
C109600	Non-insulin-dependent diabetes mellitus with retinopa
C109611	Type II diabetes mellitus with retinopathy
C109612	Type 2 diabetes mellitus with retinopathy
C109700	Non-insulin dependent diabetes mellitus - poor contro
C109711	Type II diabetes mellitus - poor control
C109712	Type 2 diabetes mellitus - poor control

C109900	Non-insulin-dependent diabetes mellitus without compl
C109911	Type II diabetes mellitus without complication
C109912	Type 2 diabetes mellitus without complication
C109A00	Non-insulin dependent diabetes mellitus with mononeur
C109A11	Type II diabetes mellitus with mononeuropathy
C109A12	Type 2 diabetes mellitus with mononeuropathy
C109B00	Non-insulin dependent diabetes mellitus with polyneur
C109B11	Type II diabetes mellitus with polyneuropathy
C109B12	Type 2 diabetes mellitus with polyneuropathy
C109C00	Non-insulin dependent diabetes mellitus with nephropa
C109C11	Type II diabetes mellitus with nephropathy
C109C12	Type 2 diabetes mellitus with nephropathy
C109D00	Non-insulin dependent diabetes mellitus with hypoglyc
C109D11	Type II diabetes mellitus with hypoglycaemic coma
C109D12	Type 2 diabetes mellitus with hypoglycaemic coma
C109E00	Non-insulin depend diabetes mellitus with diabetic ca
C109E11	Type II diabetes mellitus with diabetic cataract
C109E12	Type 2 diabetes mellitus with diabetic cataract
C109F00	Non-insulin-dependent d m with peripheral angiopath
C109F11	Type II diabetes mellitus with peripheral angiopathy
C109F12	Type 2 diabetes mellitus with peripheral angiopathy
C109G00	Non-insulin dependent diabetes mellitus with arthropa
C109G11	Type II diabetes mellitus with arthropathy
C109G12	Type 2 diabetes mellitus with arthropathy
C109H00	Non-insulin dependent d m with neuropathic arthropath
C109H11	Type II diabetes mellitus with neuropathic arthropath
C109H12	Type 2 diabetes mellitus with neuropathic arthropathy
C109J00	Insulin treated Type 2 diabetes mellitus
C109J11	Insulin treated non-insulin dependent diabetes mellit
C109J12	Insulin treated Type II diabetes mellitus
C109K00	Hyperosmolar non-ketotic state in type 2 diabetes mel
C10A.00	Malnutrition-related diabetes mellitus
C10A000	Malnutrition-related diabetes mellitus with coma
C10A100	Malnutrition-related diabetes mellitus with ketoacido
C10A200	Malnutrition-related diabetes mellitus with renal com
C10A300	Malnutrit-related diabetes mellitus wth ophthalmic co
C10A400	Malnutrition-related diabetes mellitus wth neuro comp
C10A500	Malnutritn-relat diabetes melitus wth periph circul c
C10A600	Malnutrition-related diabetes mellitus with multiple
C10A700	Malnutrition-related diabetes mellitus without compli
C10AW00	Malnutrit-related diabetes mellitus with unspec compl
C10AX00	Malnutrit-relat diabetes mellitus with other spec com
C10B.00	Diabetes mellitus induced by steroids
C10B000	Steroid induced diabetes mellitus without complicatio
C10C.00	Diabetes mellitus autosomal dominant
C10D.00	Diabetes mellitus autosomal dominant type 2
C10D.11	Maturity onset diabetes in youth type 2
C10F.00Type 2 diabetes mellitus	
C10F.11Type II diabetes mellitus	
C10F000	Type 2 diabetes mellitus with renal complications
C10F011	Type II diabetes mellitus with renal complications

C10F100	Type 2 diabetes mellitus with ophthalmic complication
C10F111	Type II diabetes mellitus with ophthalmic complicatio
C10F200	Type 2 diabetes mellitus with neurological complicati
C10F211	Type II diabetes mellitus with neurological complicat
C10F300	Type 2 diabetes mellitus with multiple complications
C10F311	Type II diabetes mellitus with multiple complications
C10F400	Type 2 diabetes mellitus with ulcer
C10F411	Type II diabetes mellitus with ulcer
C10F500	Type 2 diabetes mellitus with gangrene
C10F511	Type II diabetes mellitus with gangrene
C10F600	Type 2 diabetes mellitus with retinopathy
C10F611	Type II diabetes mellitus with retinopathy
C10F700	Type 2 diabetes mellitus - poor control
C10F711	Type II diabetes mellitus - poor control
C10F900	Type 2 diabetes mellitus without complication
C10F911	Type II diabetes mellitus without complication
C10FA00	Type 2 diabetes mellitus with mononeuropathy
C10FA11	Type II diabetes mellitus with mononeuropathy
C10FB00	Type 2 diabetes mellitus with polyneuropathy
C10FB11	Type II diabetes mellitus with polyneuropathy
C10FC00	Type 2 diabetes mellitus with nephropathy
C10FC11	Type II diabetes mellitus with nephropathy
C10FD00	Type 2 diabetes mellitus with hypoglycaemic coma
C10FD11	Type II diabetes mellitus with hypoglycaemic coma
C10FE00	Type 2 diabetes mellitus with diabetic cataract
C10FE11	Type II diabetes mellitus with diabetic cataract
C10FF00	Type 2 diabetes mellitus with peripheral angiopathy
C10FF11	Type II diabetes mellitus with peripheral angiopathy
C10FG00	Type 2 diabetes mellitus with arthropathy
C10FG11	Type II diabetes mellitus with arthropathy
C10FH00	Type 2 diabetes mellitus with neuropathic arthropathy
C10FH11	Type II diabetes mellitus with neuropathic arthropath
C10FJ00	Insulin treated Type 2 diabetes mellitus
C10FJ11	Insulin treated Type II diabetes mellitus
C10FK00	Hyperosmolar non-ketotic state in type 2 diabetes mel
C10FK11	Hyperosmolar non-ketotic state in type II diabetes me
C10FL00	Type 2 diabetes mellitus with persistent proteinuria
C10FL11	Type II diabetes mellitus with persistent proteinuria
C10FM00	Type 2 diabetes mellitus with persistent microalbumin
C10FM11	Type II diabetes mellitus with persistent microalbumi
C10FN00	Type 2 diabetes mellitus with ketoacidosis
C10FN11	Type II diabetes mellitus with ketoacidosis
C10FP00	Type 2 diabetes mellitus with ketoacidotic coma
C10FP11	Type II diabetes mellitus with ketoacidotic coma
C10FQ00	Type 2 diabetes mellitus with exudative maculopathy
C10FQ11	Type II diabetes mellitus with exudative maculopathy
C10FR00	Type 2 diabetes mellitus with gastroparesis
C10FR11	Type II diabetes mellitus with gastroparesis
C10FS00	Maternally inherited diabetes mellitus
C10G.00	Secondary pancreatic diabetes mellitus
C10G000	Secondary pancreatic diabetes mellitus without compli

C10H.00	Diabetes mellitus induced by non-steroid drugs
C10H000	DM induced by non-steroid drugs without complication
C10J.00	Insulin autoimmune syndrome
C10J000	Insulin autoimmune syndrome without complication
C10K.00	Type A insulin resistance
C10K000	Type A insulin resistance without complication
C10M.00	Lipoatrophic diabetes mellitus
C10M000	Lipoatrophic diabetes mellitus without complication
C10N.00	Secondary diabetes mellitus
C10N000	Secondary diabetes mellitus without complication
C10N100	Cystic fibrosis related diabetes mellitus
C10y.00	Diabetes mellitus with other specified manifestation
C10y000	Diabetes mellitus, juvenile, + other specified manife
C10y100	Diabetes mellitus, adult, + other specified manifesta
C10yy00	Other specified diabetes mellitus with other spec com
C10yz00	Diabetes mellitus NOS with other specified manifestat
C10z.00	Diabetes mellitus with unspecified complication
C10z000	Diabetes mellitus, juvenile type, + unspecified compl
C10z100	Diabetes mellitus, adult onset, + unspecified complic
C10zy00	Other specified diabetes mellitus with unspecified co
C10zz00	Diabetes mellitus NOS with unspecified complication
Cyu2.00	[X]Diabetes mellitus
Cyu2000	[X]Other specified diabetes mellitus
Cyu2100	[X]Malnutrit-relat diabetes mellitus with other spec
Cyu2200	[X]Malnutrit-related diabetes mellitus with unspec co
Cyu2300	[X]Unspecified diabetes mellitus with renal complicat
F171100	Autonomic neuropathy due to diabetes
F35z000	Diabetic mononeuritis NOS
F372.00	Polyneuropathy in diabetes
F372.11	Diabetic polyneuropathy
F372.12	Diabetic neuropathy
F372000	Acute painful diabetic neuropathy
F372100	Chronic painful diabetic neuropathy
F372200	Asymptomatic diabetic neuropathy
F381300	Myasthenic syndrome due to diabetic amyotrophy
F381311	Diabetic amyotrophy
F3y0.00	Diabetic mononeuropathy
F420.00	Diabetic retinopathy
F420000	Background diabetic retinopathy
F420100	Proliferative diabetic retinopathy
F420200	Preproliferative diabetic retinopathy
F420300	Advanced diabetic maculopathy
F420400	Diabetic maculopathy
F420500	Advanced diabetic retinal disease
F420600	Non proliferative diabetic retinopathy
F420700	High risk proliferative diabetic retinopathy
F420800	High risk non proliferative diabetic retinopathy
F420z00	Diabetic retinopathy NOS
F440700	Diabetic iritis
F464000	Diabetic cataract
G73y000	Diabetic peripheral angiopathy

K01x100	Nephrotic syndrome in diabetes mellitus
K08yA00	Proteinuric diabetic nephropathy
K08yA11	Clinical diabetic nephropathy
K27y700	Erectile dysfunction due to diabetes mellitus
Kyu0300	[X]Glomerular disorders in diabetes mellitus
L180600	Pre-existing diabetes mellitus, non-insulin-dependent
L180700	Pre-existing malnutrition-related diabetes mellitus
L180X00	Pre-existing diabetes mellitus, unspecified
Lyu2900	[X]Pre-existing diabetes mellitus, unspecified
M037200	Cellulitis in diabetic foot
M271000	Ischaemic ulcer diabetic foot
M271100	Neuropathic diabetic ulcer - foot
M271200	Mixed diabetic ulcer - foot
N030000	Diabetic cheiroarthropathy
N030011	Diabetic cheiropathy
N030100	Diabetic Charcot arthropathy
Q441.00	Neonatal diabetes mellitus
R054200	[D]Gangrene of toe in diabetic
R054300	[D]Widespread diabetic foot gangrene
SL23z00	Insulins or antidiabetic poisoning NOS
TJ23.00	Adverse reaction to insulins and antidiabetic agents
TJ23z00	Adverse reaction to insulins and antidiabetic agents
U602311	[X] Adverse reaction to insulins and antidiabetic age
U60231E	[X] Adverse reaction to insulins and antidiabetic age
ZC2C800	Dietary advice for diabetes mellitus
ZC2C911	Diet advice for insulin-dependent diabetes
ZC2CA00	Dietary advice for type II diabetes
ZC2CA11	Dietary advice non-insulin-dependent diabetes
ZL22500	Under care of diabetic liaison nurse
ZL62500	Referral to diabetes nurse
ZL62600	Referral to diabetic liaison nurse
ZLA2500	Seen by diabetic liaison nurse
ZLD7500	Discharge by diabetic liaison nurse
ZRB4.00	Diabetes clinic satisfaction questionnaire
ZRB4.11	CSQ - Diabetes clinic satisfaction questionnaire
ZRB5.00	Diabetes treatment satisfaction questionnaire
ZRB5.11	DTSQ - Diabetes treatment satisfaction questionnaire
ZRB6.00	Diabetes wellbeing questionnaire
ZRB6.11	DWBQ - Diabetes wellbeing questionnaire
ZRbH.00	Perceived control of insulin-dependent diabetes
ZV65312	[V]Dietary counselling in diabetes mellitus
ZV6DA00	[V]Admitted for commencement of insulin
ZV6DB00	[V]Admitted for conversion to insulin

ahdcode

1009100000	diabetes annual check
1009111000	diabetes current status
1009120000	diabetes insulin dosage
1001400140	hb a1c - diabetic control
1001400327	diabetic retinopathy screening

Prediabetes Readcodes

Review of impaired glucose tolerance

Referral for management of impaired glucose tolerance

Referral for impaired glucose tolerance management offered

Impaired glucose tolerance monitoring administration

Impaired glucose tolerance monitoring invitation

Impaired glucose tolerance monitoring invitation 1st letter

Impaired glucose tolerance monitoring invitation 2nd letter

Impaired glucose tolerance monitoring invitation 3rd letter

Impaired glucose regulation monitoring invitation

Impaired glucose regulation monitoring invitation 1st letter

Impaired glucose regulation monitoring invitation 2nd letter

Impaired glucose regulation monitoring invitation 3rd letter

Impaired glucose regulation monitoring telephone invitation

Impaired glucose regulation monitoring verbal invitation

Impaired glucose tolerance

Impaired fasting glycaemia

Impaired glucose regulation

Pre-diabetes

Non-diabetic hyperglycaemia

[D]Glucose tolerance test abnormal

[D]Prediabetes

[D]Impaired fasting glycaemia

[D]Impaired fasting glucose

[D]Impaired glucose tolerance

[X]Hyperglycaemia, unspecified

Glucose tol. test impaired

Supplementary table 1 Incidence rate of diagnosis of type 2 diabetes per 1000 PYAR in Men by calendar year

	Age 40-49		Age 50-59		Age 60-69		Age 70-79		Age 80-89		Age 90-99	
Year	Men	Women	Men	Women	Men	Women	Men	Women	Men	Women	Men	Women
2009	4.73	3.63	9.42	6.64	13.37	9.57	15.28	12.51	11.22	9.78	6.19	4.99
2010	4.87	3.73	9.3	6.62	13.65	9.35	15.26	12.14	11.42	9.35	7.44	6.28
2011	4.95	3.84	9.22	6.54	13.32	9.22	14.38	12.21	11.03	9.38	7.75	6.11
2012	4.85	3.65	9.39	6.61	13.39	9.35	14.51	11.93	11.92	10.42	8.49	6.15
2013	4.84	4.04	9.18	6.73	13.68	9.45	15.44	12.89	12.42	10.41	8.99	6.49
2014	4.57	3.31	7.94	5.94	10.92	7.97	12.36	9.54	10.87	8.53	7.24	4.99
2015	4.80	3.59	8.27	6.25	11.64	8.15	12.75	10.28	11.49	9.35	8.24	5.76
2016	4.66	3.58	7.97	6.24	11.35	8.14	11.95	9.46	10.56	8.24	7.84	4.93
2017	4.62	3.16	8.14	5.78	11.04	7.00	11.18	8.77	9.70	7.66	5.62	5.83
2018	4.20	2.88	6.60	4.60	8.91	5.86	9.47	7.09	8.48	6.5	5.48	4.09
Drop from 2013 (%)	13.22	28.71	28.10	31.65	34.87	37.99	38.67	45.00	31.72	37.56	39.04	36.98

Pal K, *et al. BMJ Open Diab Res Care* 2021; 9:e001989. doi: 10.1136/bmjdrc-2020-001989

Supplementary table 2 Incidence rates per 1000 PYAR of Prediabetes 2009-2018 in men and women using Read codes or HbA1c between 6.0-6.4% (42-47 mmol/mol)

**Year**	Men			Women		
	Prediabetes Read codes	Read codes OR ↑HbA1c	%age coded	Prediabetes Read codes	Read codes OR ↑HbA1c	%age coded
2009	2.18	3.41	63.93	1.88	3.06	61.44
2010	2.12	3.67	57.77	1.74	3.33	52.25
2011	2.05	4.06	50.49	1.69	3.76	44.95
2012	2.02	5.6	36.07	1.71	5.53	30.92
2013	2.46	8.27	29.75	2.30	8.91	25.81
2014	2.20	7.54	29.18	2.22	8.45	26.27
2015	3.26	9.61	33.92	3.46	10.59	32.67
2016	3.94	8.86	44.47	4.44	10.00	44.40
2017	3.90	7.95	49.06	4.12	8.82	46.71
2018	4.62	9.89	46.71	4.95	10.75	46.05

Pal K, *et al. BMJ Open Diab Res Care* 2021; 9:e001989. doi: 10.1136/bmjdrc-2020-001989
